# Validation of a Synthetic Wastewater Matrix as a Proxy for Municipal Wastewater for In Vitro Studies of *E. coli*

**DOI:** 10.1111/1758-2229.70450

**Published:** 2026-10-23

**Authors:** Indorica Sutradhar, Neila Gross, Carly Ching, Yanina Nahum, Darash Desai, Devin A. Bowes, Muhammad H. Zaman

**Affiliations:** ^1^ Department of Biomedical Engineering Boston University Boston Massachusetts USA; ^2^ Department of Materials Science and Engineering Boston University Boston Massachusetts USA; ^3^ Center for Forced Displacement Boston University Boston Massachusetts USA; ^4^ Department of Environmental Health Sciences, Arnold School of Public Health University of South Carolina Columbia South Carolina USA; ^5^ Center on Emerging Infectious Diseases Research Boston University Boston Massachusetts USA; ^6^ Howard Hughes Medical Institute Boston University Boston Massachusetts USA

## Abstract

Antimicrobial resistance (AMR) is a major threat to global health and resistant bacterial populations have been observed to develop and spread in and around wastewater. However, in vitro studies on AMR development are typically conducted in ideal rich media conditions, which can differ in composition and nutrient density from wastewater. In this study, we compare the growth and AMR development of *E. coli* in a synthetic wastewater recipe to autoclaved wastewater samples from the Massachusetts Water Resources Authority (MWRA). We found that synthetic wastewater and real wastewater samples both supported less bacterial growth compared to LB broth and had similar susceptibility profiles to antibiotic pressure from Doxycycline, Ciprofloxacin and Streptomycin. More importantly, unlike in LB broth, AMR development over time during continuous passaging under subinhibitory antibiotic pressure in pH‐adjusted synthetic wastewater was similar in fold change to real wastewater samples. Thus, we suggest that synthetic wastewater is a more accurate predictor of both *E. coli* growth and antibiotic resistance development in wastewater systems than standard nutrient broths such as LB. The validation of synthetic wastewater as a better proxy for real wastewater will allow for further recapitulation of the wastewater environment in future in vitro studies of AMR in these conditions.

## Graphical abstract

In vitro studies of antimicrobial resistance (AMR) usually use rich laboratory media that differ from environmental conditions. We compared *E. coli* growth, antibiotic susceptibility and resistance development in LB broth, synthetic wastewater and autoclaved municipal wastewater. Both wastewaters restricted growth relative to LB and pH‐matched synthetic wastewater reproduced the susceptibility and resistance patterns seen in real wastewater, supporting its use as a proxy in AMR studies.

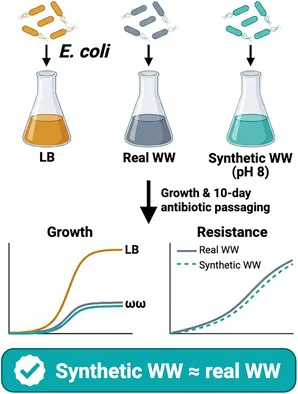

## 1 Introduction

Antibiotics and antimicrobials are essential medicines to treat infectious diseases. However, when bacteria gain antimicrobial resistance (AMR), these medicines no longer work, threatening humans, animals and the environment. Our understanding of the role of the environment in AMR is starting to increase. Wastewater is a key component of a larger and more complex shared environment in which AMR can spread and develop (UNEP 2021).

Wastewater is a major reservoir of AMR due to the collection of antibiotic pollution from inappropriate drug disposal and effluent from pharmaceutical manufacturers, hospitals and agricultural/veterinary settings (Nadimpalli et al. 2020). In some cases, environmental concentrations can reach, or even exceed, minimal inhibitory concentrations of certain antibiotics (Bengtsson‐Palme and Larsson 2016). This problem is particularly pertinent in low and middle‐income countries (LMICs), where cases of antibiotic‐resistant infections have been rising and 70% of sewage produced is estimated to enter the environment untreated (Nadimpalli et al. 2020). Antibiotic pollution is a major driver of resistance, as selective pressure from antibiotics in the environment is known to promote chromosomal resistance mutations (Bengtsson‐Palme and Larsson 2016; Ching and Zaman 2020). These antibiotics, as well as disinfectants in sewage, may also be able to support and promote horizontal transfer of resistance genes among bacteria (Lopatkin et al. 2016) and such mobile resistance genes have been measured at high levels in wastewater and sewage (Hastings et al. 2004). Though AMR and its drivers have been studied in sewage and wastewater settings to some extent (Baker et al. 2016; Birkegård et al. 2018), one of the largest gaps in understanding the emergence of AMR within a sewage environment is the limited understanding of the effects and interactions of the many biological and environmental mechanisms at work due to the complexity of wastewater (Brinch et al. 2020).

Aside from antibiotics, wastewater is a complex matrix that contains a mixture of a variety of substances as a result of human, animal and plant activity within the built and natural environments. Composition changes depending on the immediate local conditions that determine the sources of the constituents. However, generally all wastewater types contain water, organic matter, organic chemicals, nutrients, metals and other inorganic materials and microorganisms (mainly fungi, bacteria, protozoa and viruses) (Chahal et al. 2016). Wastewater treatment plants are widely recognized as potential hotspots of conferring AMR due to this inherent complex mixture. Additionally, other factors such as population growth, increased use and distribution of antibiotics and products from human activity such as metal‐based pesticides, fertilizer runoff from agricultural practices and persistence of toxic chemicals from personal care products all contribute to promoting favourable conditions for the growth and transfer of resistance genes within the generalized wastewater treatment infrastructure (Novo et al. 2013). Increased contamination of heavy metals from industrial runoff into the surrounding environment has encouraged microorganisms to evolve mechanisms of co‐resistance, enabling them to tolerate higher concentrations of both heavy metals and antibiotics (Edet et al. 2023). This has become of particular concern from a global health perspective in regions where heavy metal accumulation in the environment is a result of lack of treatment facilities that support proper management of waste and other related activities, providing fruitful opportunities for co‐resistance to occur over prolonged periods of time (Edet et al. 2023).

Given the complex and variable composition of wastewater in the environment, robust in vitro investigation can help us better understand which components—or combinations and concentrations—contribute to enhanced AMR. However, current laboratory‐based AMR studies are typically performed in nutrient‐rich media, which do not accurately represent the composition and concentrations present in wastewater. In contrast, it has been shown that bacteria grown in wastewater systems have altered characteristics, including changes in cell envelope composition and morphology (Chai 1983; Hirsch 1986; Rajkowski and Rice 1999). In addition, nutrient limitations found in wastewater can serve as environmental stressors that can lead to biofilm formation and the development of AMR (Burdon et al. 2020). Thus, experiments and models with rich‐nutrient media may not accurately represent AMR dynamics in wastewater. While several standard synthetic wastewater recipes have been developed to perform wastewater studies (OECD Guidelines for the Testing of Chemicals 2001; Kargol et al. 2023; Aiyuk and Verstraete 2004; Boeije et al. 1999), these have not been applied widely to bacterial or AMR studies. Moreover, there is limited evidence on how bacteria behave in real wastewater samples (Hutinel et al. 2021). To address this gap, we seek to validate the use of synthetic wastewater as a realistic proxy for wastewater when characterizing bacterial growth, antibiotic susceptibility and AMR development. Developing accurate experimental methodologies is a critical step in understanding current interactions between environmental exposures and bacteria that contribute to AMR, as it can help inform more effective strategies to combat AMR in natural and engineered settings.

## 2 Methods

### 2.1 Strains and Media Conditions

*E. coli* MG1655 (ATCC 700926) was used for all experiments. LB broth was used for the control media. Synthetic wastewater was composed according to OECD guidelines with the components listed in Table 1 mixed in 1 L of deionized water (OECD Guidelines for the Testing of Chemicals 2001; Kargol et al. 2023). Using this base formulation, we prepared solutions at 1×, 5× and 25× concentrations to evaluate the effects of varying wastewater compositions.

**TABLE 1:** Components and quantities of synthetic wastewater per 1 L DI water (OECD Guidelines for the Testing of Chemicals 2001; Kargol et al. 2023).

Component	Quantity
Peptone	160 mg
Meat extract	110 mg
Urea	30 mg
Anhydrous dipotassium hydrogen phosphate (K_2_HPO_4_)	28 mg
Sodium chloride (NaCl)	7 mg
Calcium chloride dihydrate (CaCl_2_·2H_2_O)	4 mg
Magnesium sulfate heptahydrate (MgSO_4_·7H_2_O)	2 mg

### 2.2 Wastewater Sampling

Twenty‐four hours‐composite wastewater ‘influent’ samples from the Massachusetts Water Resource Authority (MWRA) were collected into 2 L‐bottles and sealed for pick up.

Wastewater undergoes primary treatment, where grit is removed and disposed of at landfills and clarifiers are used to remove pollutants, it then moves to the secondary treatment where mixers, reactors and clarifications remove remaining solids through biological and gravitational processes (MWRA 2024). ‘Effluent’ samples were collected following this procedure. Samples were then collected from the Wastewater treatment plant (WWTP) and immediately autoclaved on a liquid cycle at 121°C for 45 min.

### 2.3 Bacterial Growth in Synthetic Wastewater and Autoclaved Real‐Wastewater Samples

To monitor growth, OD_600_ was measured every 5 min for 48 h using a Biotek plate reader with shaking in between each measurement. Wells were seeded with exponential phase wild type *E. coli* MG1655 such that the starting OD_600_ of each well was ~0.08–0.09. To avoid condensation at 37°C we made the plate cover hydrophilic as previously described (Brewster 2003).

To measure viable cell growth, wild‐type *E. coli* MG1655 was cultured at 37°C in 4 mL of the media of interest in culture tubes and sampled at 0, 1 and 24 h. These samples were plated in triplicate on LB agar to determine the CFU/mL at the time point of interest.

### 2.4 MIC in Synthetic Wastewater and Autoclaved Real‐Wastewater Samples

Doxycycline, ciprofloxacin and streptomycin were selected to represent three clinically important antibiotic classes with distinct mechanisms of action: a tetracycline (doxycycline) and an aminoglycoside (streptomycin), both of which inhibit protein synthesis at the 30S ribosomal subunit through different mechanisms and a fluoroquinolone (ciprofloxacin), which inhibits DNA gyrase and topoisomerase IV. Wild‐type *E. coli* MG1655 cultured at 37°C in the media of interest was grown in 96‐well plates with 2‐fold increments of ciprofloxacin, doxycycline, or streptomycin. Each media condition was run in biological triplicates (*n* = 3). The minimum inhibitory concentration (MIC) was determined to be the lowest concentration of antibiotic at which no growth was observed, as determined by OD measurement. OD measurements were also conducted in control wells with no seeded bacteria to determine interference from suspended solids in autoclaved real‐wastewater samples.

### 2.5 Rate of Resistance Development of *E. coli* in Synthetic Wastewater and Autoclaved Real‐Wastewater Samples

Wild‐type *E. coli* MG1655 cultured at 37°C in the media of interest was grown in 96‐well plates with 2‐fold increments of ciprofloxacin or doxycycline. Each media condition was run in biological triplicates (*n* = 3). We selected the bacteria in the well closest to 50% of the inhibitory concentration (IC50) to seed new bacterial cultures on the same dose series of ciprofloxacin at ~24 h, for 10 days. Bacteria serially passaged in LB broth media for the duration of the experiments served as the control groups.

### 2.6 Characterization of Wastewater Samples

pH of each wastewater sample was tested using Cytiva pH test strips. Organic matter and ammonium content were measured using COD digestion vials (kit 2125825, Hach Company) and N‐Ammonia Reagent Set (kit 2606945, Hach Company) test kits and concentrations were measured with a Hach DR900 Colorimeter (Hach Company, Loveland, CO, US).

## 3 Results

### 3.1 Characterization of Different Media and Wastewater

We characterized relevant properties of LB, synthetic wastewater and autoclaved real‐wastewater samples, including pH and important macro and micronutrients. The pH, the concentration of organic matter–measured as COD— and ammonium (NH_3_—N) concentration for either real wastewater samples, synthetic wastewater and LB media can be observed in Table 2. The synthetic wastewater sample exhibited the lowest ammonium concentration, which could potentially limit growth. On the contrary, LB media contained the highest concentration of NH_3_‐N and organic matter, providing optimal nutrient conditions for robust growth. The influent and effluent autoclaved real‐wastewater samples showed average concentrations of both NH_3_—N and COD, comparable to other mainstream wastewater sources, which contain between 20–60 mg N/L of ammonia and typical concentrations of COD below 1000 mg/L (Gilbert et al. 2014; Mergaert et al. 1992).

**TABLE 2:** Sample characterization.

	Autoclaved wastewater—influent	Autoclaved wastewater—effluent	Synthetic wastewater (1×)	LB
pH	8	8	6	7
COD (mg/L)	155	43	289	16,600
NH_3_ (mg‐N/L)	30	19	5	84

### 3.2 *E. coli* Display Reduced Growth in Synthetic and Real Wastewater

In order to compare the growth of *E. coli* in the synthetic wastewater and in autoclaved real‐wastewater samples to the growth in LB broth, we performed plate reader growth curve measurements (Figure 1) over 48 h. We observed reduced growth in synthetic wastewater, with the 25× synthetic wastewater recipe showing more growth than the 5× strength. The synthetic wastewater recipe at the literature concentrations (1×) had the lowest growth among synthetic wastewater recipes and similar growth to the influent. The lowest growth was observed in the effluent, which exhibited similar growth patterns to tap and DI water. We also verified these growth curves with colony counts on LB agar plates after 1 h and 24 h (Figure 2). After 1 h, all media conditions displayed similar bacterial growth. However, after 24 h, *E. coli* colony counts reflected concentrations similar to those predicted by the plate reader growth curves. The relative growth rates to *E. coli* grown in LB (Figure 2) were also reported. While all samples were statistically different from *E. coli* grown in LB, the effluent was statistically different from the influent, 25× synthetic wastewater and 5× synthetic wastewater. While the 25× synthetic wastewater was observed to have a lower OD_600_ than LB, both media conditions showed similar colony counts on agar, possible due to the presence of dead cells in the LB condition from the rapid growth. The influent showed a similar relative growth rate to the 1× synthetic wastewater.

**FIGURE 1:**
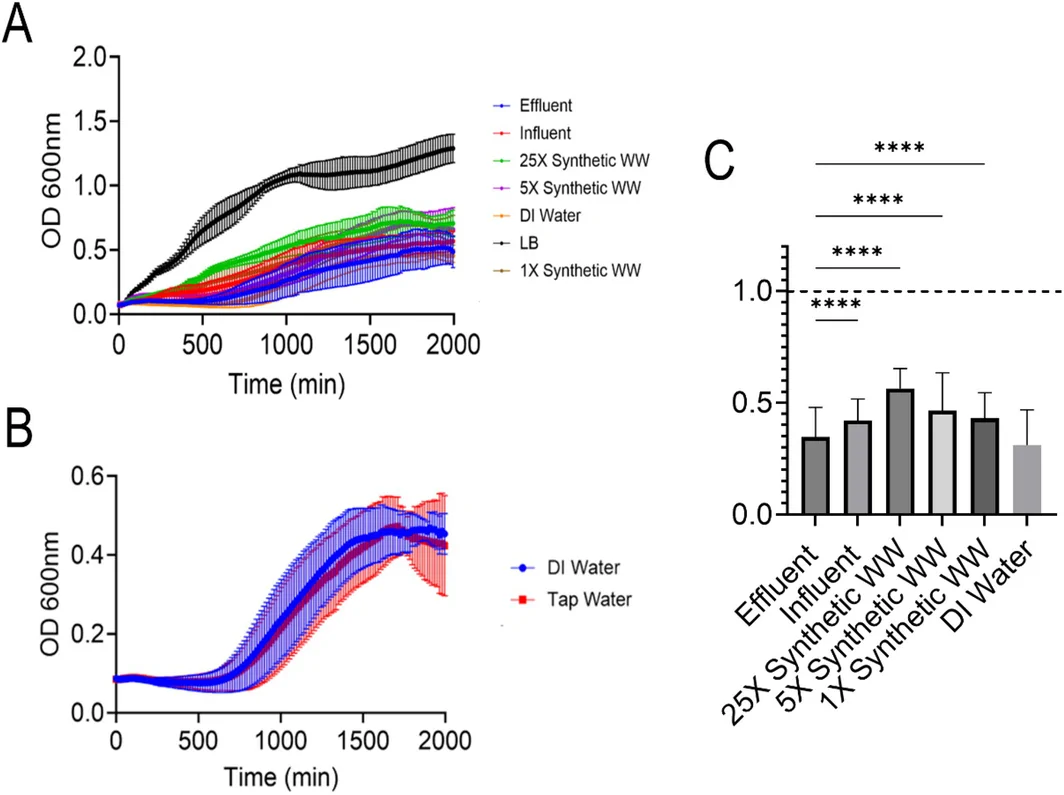
OD_600_ measurements of *E. coli* growth over 48 h at 37°C. (A) Comparison of influent and effluent autoclaved real‐WW, 1×, 5× and 25× Synthetic WW (SWW), DI Water and LB, (B) Autoclaved tap and DI water and (C) relative growth rates to *E. coli* in LB (dashed line), **** represents population STDEV statistical significance.

**FIGURE 2:**
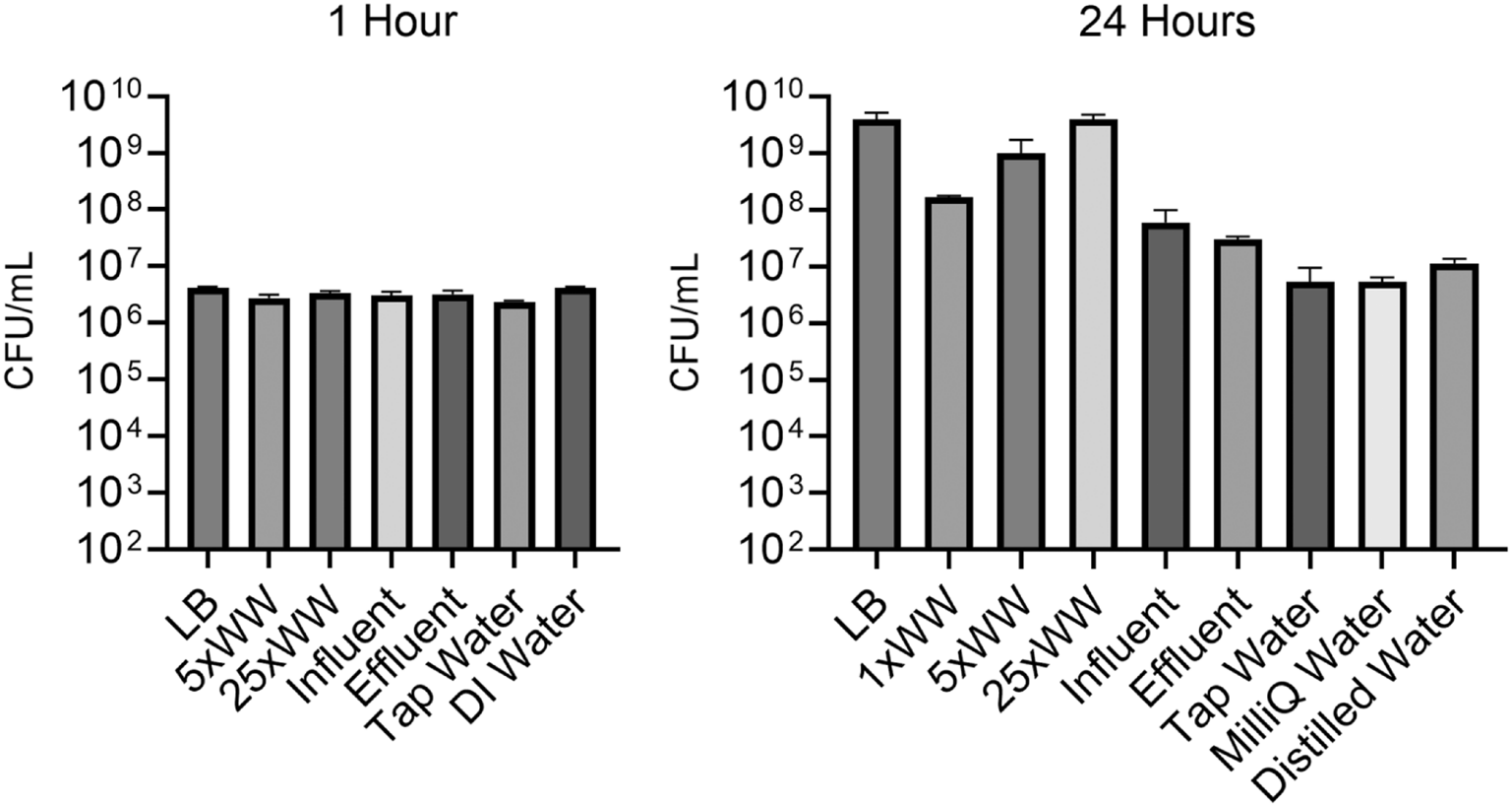
*E. coli* Colony counts on Agar Plates after 1 h and after 24 h.

### 3.3 *E. coli* Grown in Wastewater Have Altered Sensitivities to Antibiotics, Suggesting Synergistic and Antagonistic Relationships With Wastewater Components

In order to further compare the synthetic wastewater and the real‐wastewater samples to each other and to LB media, we studied antibiotic activity against *E. coli* in each of these media. We first compared the MIC of doxycycline, an antibiotic that has been previously detected in wastewater, for *E. coli* in each of the media conditions (Figure 3A). While similar MICs to doxycycline were observed in the synthetic wastewater and LB, the influent and effluent exhibited a significantly increased MIC. As one difference we noted among the different media was pH, we tested compositions with increased pH to match the basic pH of the real wastewater and observed that the synthetic wastewater with a pH of 8 and a pH of 10 achieved similarly increased MIC to the influent and effluent samples. Prior literature has shown that pH higher than 8 can decrease doxycycline absorption (Álvarez‐Esmorís et al. 2022), which is consistent with the decreased doxycycline activity observed in the autoclaved real wastewater samples and the basic synthetic wastewater.

**FIGURE 3:**
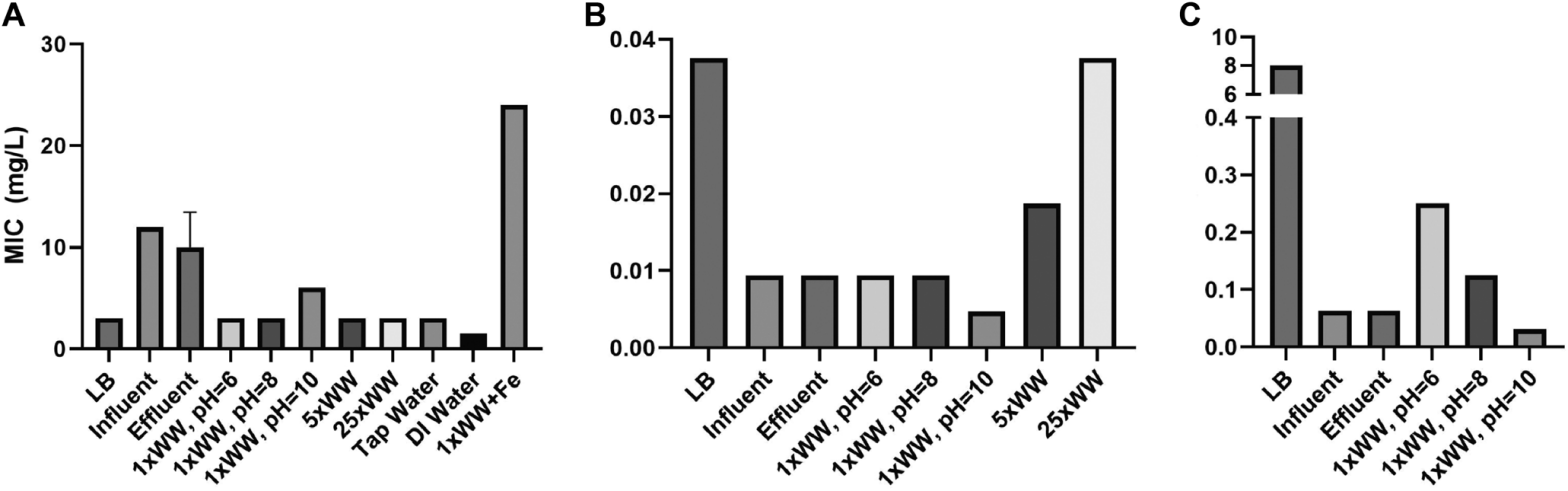
(A) Comparison of Doxycycline MICs for various media conditions. (B) Comparison of Ciprofloxacin MICs for various media conditions. (C) Comparison of Streptomycin MICs for various media conditions. LB, tap water and DI water are at pH = 7, 5× and 25× synthetic wastewater are at pH = 6 and Influent and Effluent are at pH = 8. Error bars represent standard deviation.

We next compared the MIC of ciprofloxacin for each of the media conditions (Figure 3B). In this case, the less concentrated synthetic wastewater recipes exhibited decreased MICs compared to their respective decreased bacterial concentrations. This decreased MIC was also observed for the influent and effluent samples. We performed the same MIC assay with the synthetic wastewater at various pHs and with the real wastewater for Streptomycin, showing reduced MICs at high pHs and in the influent and effluent samples of real wastewater (Figure 3C).

### 3.4 Resistance Develops in All Media, Including Real Wastewater Samples, on Similar Timescales, but Absolute Values Change Based on Different Starting MICs

To further compare antibiotic activity in the synthetic wastewater and the wastewater samples, we performed serial passaging experiments to probe how the different media affect the development of antibiotic resistance. Due to the impact of pH on the antimicrobial activity of both Doxycycline and Streptomycin, 1× synthetic wastewater was adjusted to a pH of 8 and compared to the real wastewater samples (already at pH = 8). Performing serial passaging in doxycycline (Figure 4) showed that the *E. coli* passaged in influent and effluent real wastewater and in the pH‐adjusted synthetic wastewater exhibited a lower fold change in MIC over 10 days and the absolute MIC of all conditions developed to similar levels.

**FIGURE 4:**
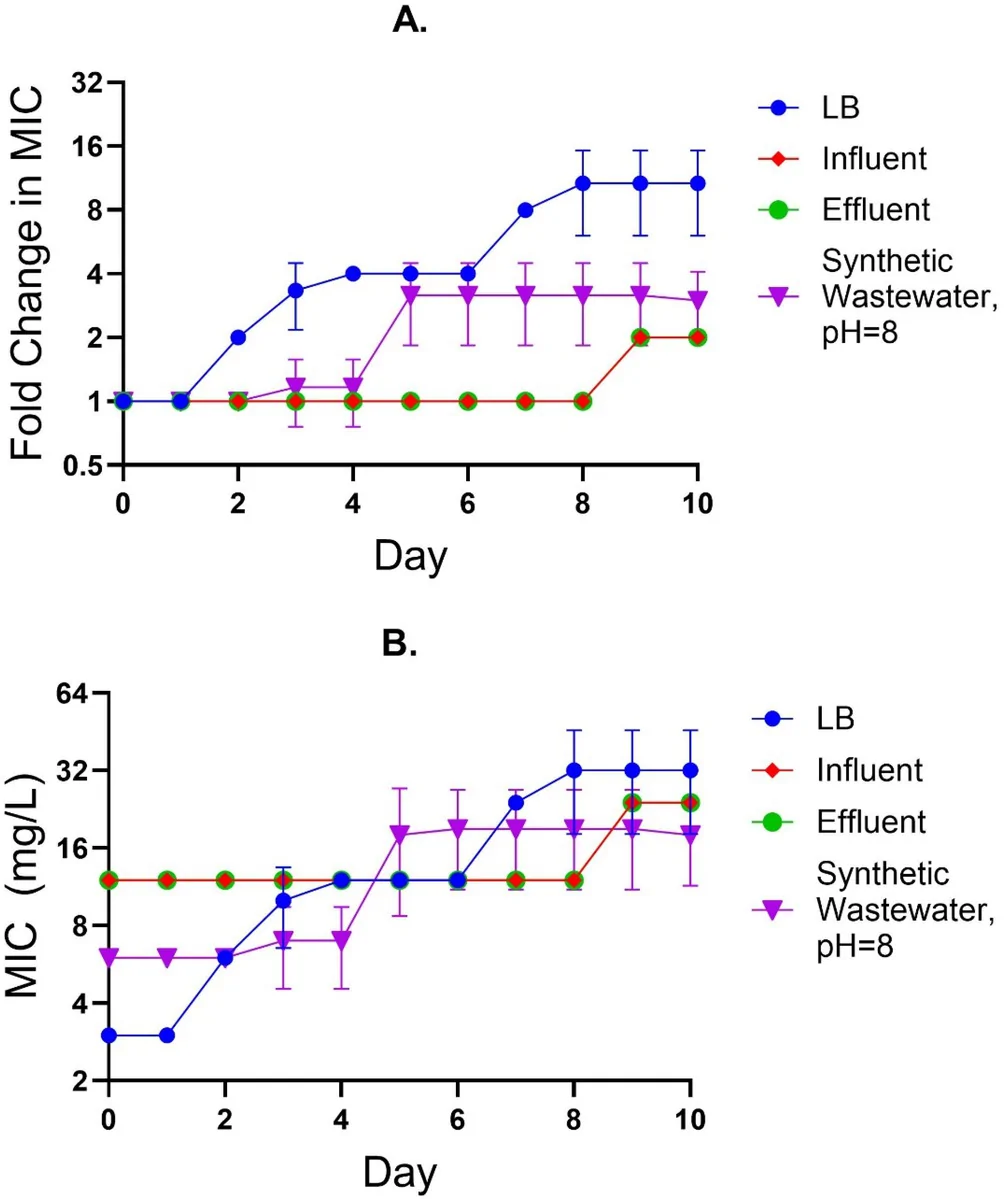
Change in Doxycycline MIC when passaged over time under different media conditions. (A) Fold change in Doxycycline MIC from baseline at Day 0. (B) Absolute MIC to Doxycycline over time, error bars represent standard deviation.

Performing serial passaging in ciprofloxacin (Figure 5) also exhibited similar behaviour between *E. coli* passaged in the influent and effluent samples and *E. coli* passaged in synthetic wastewater, both in absolute MIC over time and in fold change in MIC over time.

**FIGURE 5:**
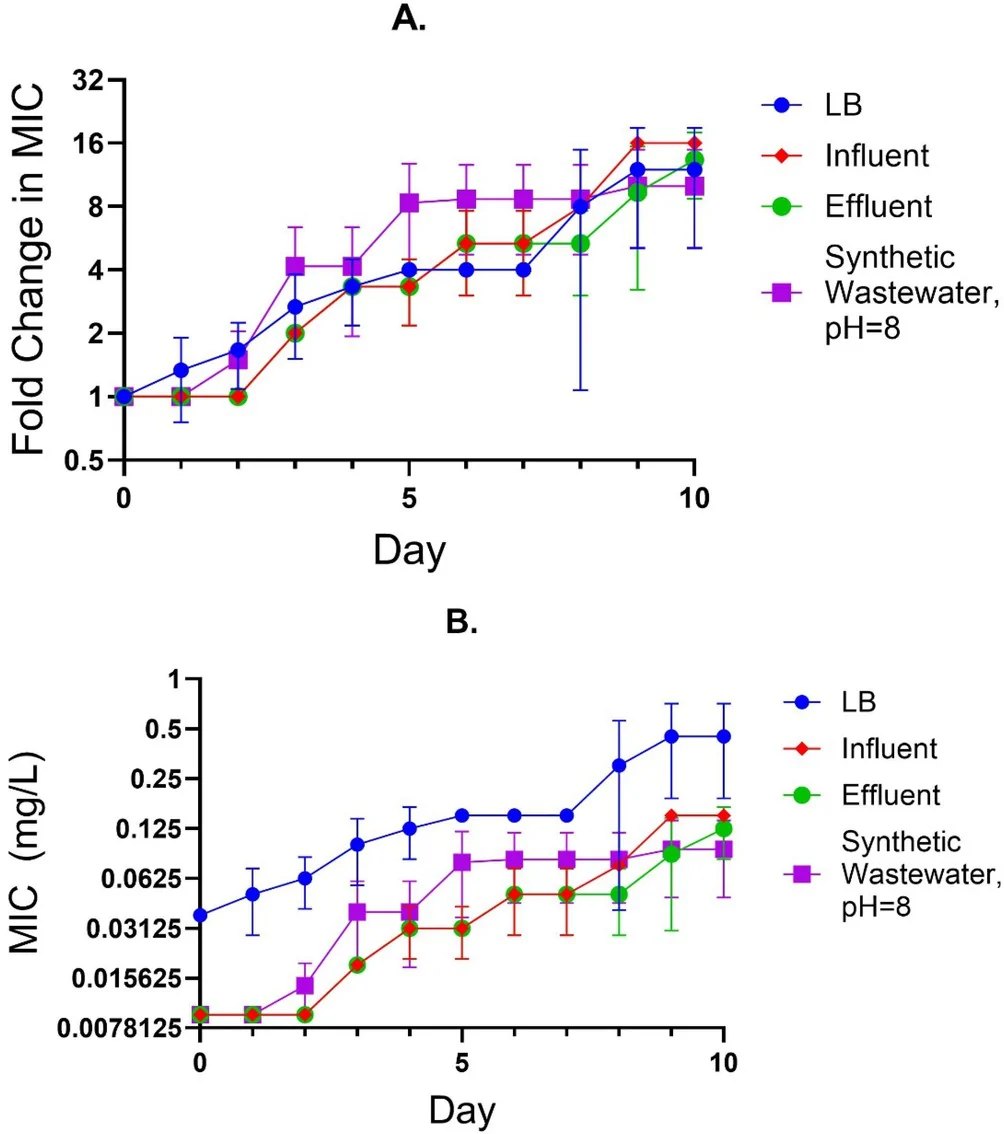
Change in Ciprofloxacin MIC when passaged over time under different media conditions. (A) Fold change in Ciprofloxacin MIC from baseline at Day 0. (B) Absolute MIC to Ciprofloxacin over time, error bars represent standard deviation.

We also repeated the serial passaging experiment with streptomycin (Figure 6). In streptomycin, the media conditions all exhibit similar fold changes in MIC over the course of the experiment, with LB exhibiting a significantly higher absolute MIC and the synthetic wastewater and wastewater samples exhibiting similarly low absolute MICs.

**FIGURE 6:**
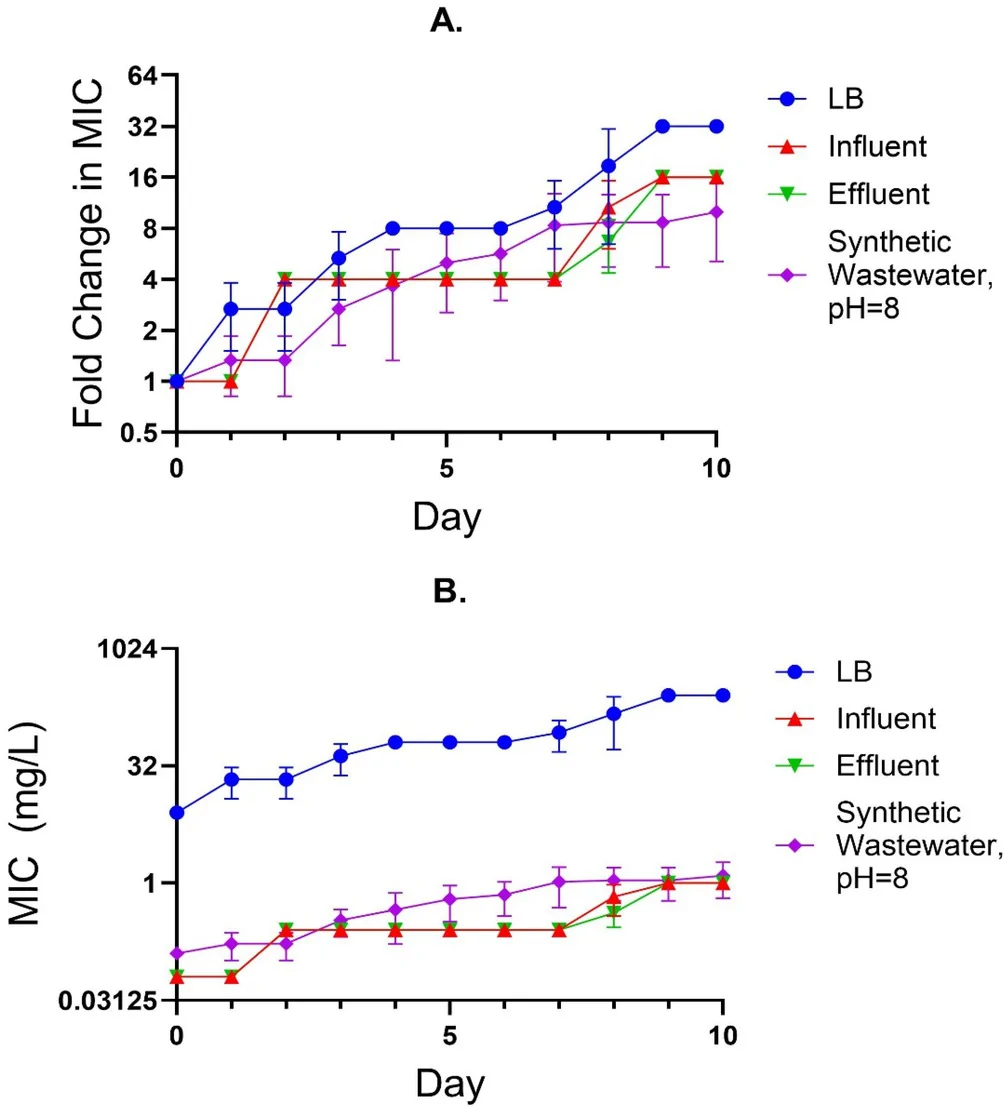
Change in Streptomycin MIC when passaged over time under different media conditions. (A) Fold change in Streptomycin MIC from baseline at Day 0. (B) Absolute MIC to Streptomycin over time, error bars represent standard deviation.

## 4 Discussion

These studies in synthetic and real wastewater samples demonstrate that synthetic wastewater can be an acceptable proxy for real wastewater in modelling the growth of *E. coli* and also the response of *E. coli* to antibiotic pressure. Both *E. coli* grown in synthetic wastewater and *E. coli* grown in autoclaved real wastewater showed reduced relative growth rate and reduced culture density after 24 h compared to growth in LB, consistent with their reduced nutrient concentration. This effect was particularly pronounced in synthetic wastewater, possibly due to the initial ammonium concentration–the most readily available nitrogen source–which was nearly at limiting concentrations from the start of the growth curve. In addition to the concentration of macronutrients, the varying concentrations of micronutrients in the real and synthetic wastewater samples–not measured in this study–may have influenced the growth rates of *E. coli* .

Additionally, the *E. coli* grown in synthetic wastewater adjusted to a pH of 8 displayed similar behaviour to the *E. coli* grown in autoclaved real wastewater samples, which had a significantly higher MIC to Doxycycline, a comparable MIC to Ciprofloxacin and a significantly lower MIC to Streptomycin as compared to *E. coli* grown in LB media. This demonstrates that pH‐adjusted synthetic wastewater can capture environmental *E. coli* behaviour in response to antibiotic pressure. Thus, we would suggest matching the pH of synthetic wastewater to the real wastewater samples of interest to best capture the effects of antibiotics on bacterial growth in these environments. Additionally, we show similar antimicrobial resistance development under subinhibitory antibiotic pressure over time in both the synthetic and autoclaved real wastewater. Studies in Doxycycline, Ciprofloxacin and Streptomycin showed similar absolute MICs over time and similar fold changes in MIC over time as compared to the initial MICs in pH‐adjusted synthetic wastewater as compared to autoclaved real wastewater samples. This indicates that synthetic wastewater that is pH matched to the wastewater of interest is a suitable proxy for real wastewater when studying antibiotic resistance development under subinhibitory antibiotic pressure.

One limitation of this study was the necessity of autoclaving the wastewater samples for use as a growth medium, which had the potential to degrade or alter some of the wastewater components. The experimental design required the use of sterile media to be comparable to lab‐made media such as LB. While we chose to autoclave the wastewater samples to maintain the larger solid particulate matter, filter sterilization could be used in future studies to better understand the composition of real wastewater. Additionally, while the low activity of Doxycycline in the wastewater samples was partially recapitulated by adjusting the pH of the synthetic wastewater, organic surfaces in the wastewater samples may have also reduced the biological availability of the antibiotic. Identifying and matching the organic compounds in wastewater samples could serve to further bring synthetic wastewater recipes to real wastewater conditions. We were also limited in the number of wastewater samples tested here, with only influent and effluent samples from one wastewater treatment plant. Further studies with samples from other geographic locations nationally and internationally could further validate the findings here and shed light on key differences between wastewater environments across different treatment conditions and climates. Additionally, real wastewater environments typically have more than one bacterial species and future studies with multiple bacterial species could further elucidate the validity of LB media and synthetic wastewater as proxies for real wastewater in in vitro studies, particularly for AMR development. Further characterization of real wastewater samples, such as the concentration of micronutrients, would also provide greater insight into developing synthetic wastewater recipes that more closely recapitulate environmental wastewater conditions.

## 5 Conclusion

Wastewater environments play a critical role in the development and spread of AMR, as resistant bacterial populations are frequently observed in these settings. However, most in vitro studies on AMR are conducted under media conditions that differ significantly from the composition and nutrient density found in actual wastewater. In our study, we observed that the use of real wastewater samples as media for in vitro assays with *E. coli* resulted in differences in growth and response to antibiotic pressure as compared to LB broth. However, by using synthetic wastewater at different strengths and at a slightly basic pH, we were able to capture these differences. Decreased growth in real wastewater was matched by decreased growth in the synthetic media correlated with decreased nutrient density in these media. By adjusting the pH of the synthetic wastewater media, we were also able to capture the differences in antibiotic susceptibility that were observed for Doxycycline, Ciprofloxacin and Streptomycin. Additionally, when observing AMR development under selective pressure from subinhibitory antibiotics, pH‐adjusted synthetic wastewater captured the behaviour of real wastewater to a significant degree, thus encouraging its use as a proxy for real wastewater in in vitro AMR studies. These findings are crucial for enhancing public health surveillance methodologies, such as wastewater surveillance, as they offer valuable insights into analysing the spread of AMR in different communities. Effective detection and monitoring of AMR can help develop public health strategies and interventions to prevent and control AMR.

## Author Contributions

**Indorica Sutradhar:** conceptualization, investigation, writing – original draft, methodology, writing – review and editing, formal analysis, data curation. **Neila Gross:** conceptualization, investigation, writing – original draft, writing – review and editing, methodology, formal analysis, data curation. **Carly Ching:** conceptualization, writing – original draft, writing – review and editing, methodology, formal analysis. **Yanina Nahum:** writing – review and editing, methodology, formal analysis. **Darash Desai:** methodology, writing – review and editing, formal analysis. **Devin A. Bowes:** conceptualization, supervision, methodology, writing – review and editing, formal analysis. **Muhammad H. Zaman:** conceptualization, funding acquisition, writing – review and editing, project administration, supervision, resources.

## Funding

This work was supported by the National Science Foundation (2023354788).

## Conflicts of Interest

The authors declare no conflicts of interest.

## Data Availability

All data used for this study has been included in the manuscript. The preprint can be found on bioRxiv.
